# The involvement of working memory during retrieval from episodic memory

**DOI:** 10.3758/s13421-025-01786-x

**Published:** 2025-10-03

**Authors:** Marton F. Kocsis, Simon Farrell

**Affiliations:** School of Psychological Science, University of Western Australia, 35 Stirling Highway, Perth, WA 6009 Australia

**Keywords:** Working memory, Episodic retrieval, Free recall, Divided attention

## Abstract

While the role of attention via WM during free recall has been well established in the dual-task literature, the potential role of storage-based aspects of WM in episodic retrieval has yet to be explored, particularly outside the context of individual differences. We enforced a storage-based WM load during the recall window of a free recall-based task to examine effects on a variety of episodic recall characteristics. We assessed the effects of the load on benchmark effects from the free-recall literature (primacy, recency, lag-recency) and the rate of memory search. While load affected overall recall accuracy, there was otherwise no reliable effect of a storage-based WM load on the benchmark effects of free recall. The impairment of recall accuracy under WM load without any commensurate effect on fundamental aspects of episodic retrieval is challenging for perspectives in which the differences in episodic retrieval uniquely arise from effects on the rate of memory search, as has usually been observed in both the dual-task and WM literatures.

Working memory (WM) is a limited-capacity form of memory primarily used for the short-term storage and manipulation of information (e.g., Baddeley & Hitch, 1974; Cowan, 1999; Daneman & Carpenter, 1980; see Cowan, 2017 for review). It has been increasingly recognized that working memory is related to episodic memory, a form of long-term memory that characterizes our ability to revisit past experiences (Tulving, 1983). Models of human memory have envisioned WM as either the short-term “primary memory” component of a dual-store model of memory where episodic memory is the primary form of long-term storage (e.g., Atkinson & Shiffrin, 1968; Raaijmakers & Shiffrin, 1980), or as a highly activated subset of information within a single, long-term memory store (e.g., Cowan, 1999; Oberauer, 2007; see also Atkinson & Shiffrin, 1971). Over the past several decades, individual differences studies have shed further light on this relationship by examining the interrelated nature of WM, long-term memory, and intelligence. These studies have reliably shown a positive relationship between the long-term memory factor associated with episodic retrieval-based tasks such as free recall and the WM factor based on tasks such as complex span (Brewer & Unsworth, 2012; Mogle et al., 2008; Shipstead, Lindsey, Marshall, & Engle, 2014; Unsworth et al., 2014; Unsworth et al., 2009; Unsworth et al., 2010; Wilhelm et al., 2013; see Unsworth, 2019 for review).

Our interest is in the role of working memory in retrieval from episodic memory. Findings from the individual differences literature point to a role for working memory in controlling search from episodic memory. For example, previous examinations of free-recall response latencies have found that the increasing cumulative response times typically found during standard episodic retrieval tasks such as free-recall and related variants (e.g., delayed free recall, Unsworth, 2007; cued recall, Unsworth et al., 2009) are well captured by cumulative form on an exponential function:

$$ F(t) = N(1- e^{-\lambda t}), $$

where $N$ represents the asymptote of the function that denotes maximal total recall, and $\lambda$ is the rate of approach to the asymptotic level of recall. Such a pattern is consistent with idea that retrieval involves items in the search set being sampled with replacement, which in combination with previously sampled and reported being suppressed results in a “pure death” process (Bousfield et al., 1954; McGill, 1963; for review see Wixted & Rohrer, 1994): As more targets are sampled and reported from the search set, the probability of the sampling any remaining unsampled targets becomes increasingly smaller. Evidence for an involvement of working memory has come from studies showing that high working memory capacity (WMC) individuals had higher estimates for both $\lambda$ and $N$ relative to low WMC individuals, arguing that these differences reflect the ability to delimit a smaller search set during retrieval that includes fewer irrelevant items, and therefore recall more target items (Unsworth & Engle, 2007).

Additional evidence for higher WMC individuals having more effectively delimited search sets comes from a study by Spillers and Unsworth (2011), who looked at temporal contiguity during free recall. Temporal contiguity refers to a phenomenon observed during free recall in which people tend to recall contiguously presented items together, typically with a tendency to forward order of recall; this is known as the ‘lag-recency effect’ (Kahana, 1996). The temporal contiguity observed during free recall is argued to reflect the fundamental role of temporal context in being able to distinguish memories for the different events in our pasts within episodic memory (e.g., Farrell, 2012; Howard & Kahana, 2002; Polyn et al., 2009; Sederberg et al., 2008). Spillers and Unsworth (2011) found that low WMC individuals also showed weaker lag-recency compared to high WMC individuals, and suggested that the weaker lag recency shown by low WMC individuals represents a poorer ability to utilize the temporal-contextual information during retrieval to delimit the search set of items in memory appropriately. Similar conclusions were drawn by Unsworth et al. (2013), who found that the poorer performance on a category fluency task by low WMC individuals could be ameliorated by explicitly providing additional contextual information (e.g., common categories of animals when trying to name as many animals as possible). Critically, providing additional contextual information to high WMC individuals did not improve performance, suggesting this information was already being used during recall.

While such individual differences research is compelling, it is limited in its ability to provide direct support for the involvement of working memory, and, more importantly, how working memory is involved. One issue is that individuals higher in WMC may more effectively encode information into episodic memory, as well as being more effective memory searchers; therefore, it is challenging to tie the individual differences in WMC specifically to retrieval uniquely.

One way in which effects can be specifically localized at encoding versus retrieval is using dual-task studies. These studies have consistently found that an attention-demanding concurrent task during encoding is more detrimental to subsequent memory performance than the same task during recall (e.g., Baddeley et al., 1984; Craik et al., 1996; Naveh-Benjamin et al., 1998). Some authors have taken this asymmetry as evidence that while encoding requires attention, the process of retrieval is largely automatic and “protected” (Naveh-Benjamin et al., 2000; 2006; though see Rohrer & Pashler, 2003). Attention is argued to be centrally involved in episodic retrieval according to both the individual differences in WMC and dual-task literatures, despite each taking different positions on whether differences at encoding (as suggested by dual-task studies such as Naveh-Benjamin et al., 2000) or retrieval (as argued by WMC studies such as Unsworth & Engle, 2007) are the primary determinants of free-recall performance.

Accordingly, our interest is specifically in how working memory capacity at retrieval affects our ability to retrieve information from episodic memory. Specifically, it is not clear whether the reduced rate of retrieval under attention-demanding concurrent tasks (Naveh-Benjamin et al., 2000; Rohrer & Pashler, 2003) is the result of an impaired ability to use temporal-contextual information to effectively drive retrieval – as implied by WMC literature (Spillers & Unsworth, 2011) – as dual-task studies have not used more diagnostic measures of temporal context involvement such as temporal contiguity. There is also a further question of how the storage (i.e., maintenance) aspect of WM may be involved in episodic retrieval. While models such as that of Unsworth and Engle (2007) emphasize the role of controlled search from secondary memory in working memory capacity, a converse question is about the role of attention versus storage aspects of working memory in the task of retrieval itself (as measured by the free-recall task). While the relationship between attention and both WM (e.g., Chow & Conway, 2015; Shipstead et al., 2012; Unsworth & Brewer, 2010) and episodic retrieval (e.g., Kane & Engle, 2000; Naveh-Benjamin et al., 2000; Rohrer & Pashler, 2003; Unsworth & Engle, 2007) has been extensively examined, the potential role of the storage component of WM during episodic retrieval has not been examined.

One specific potential role of the storage component of WM during episodic retrieval is to keep track of what items have been retrieved, thereby minimizing repetitions (i.e., reporting the same item twice). A common finding of the free-recall literature is that people commit few repetitions (Lohnas et al., 2015; Unsworth et al., 2010, 2012; Zaromb et al., 2006). Models of episodic memory often assume some mechanism for preventing erroneous repetitions (e.g., resampled items that have already been recalled being suppressed, Raaijmakers & Shiffrin, 1981), particularly sampling-with-replacement models in which people explicitly filter out resamples of already recalled items(Unsworth & Engle, 2007; Wixted , Rohrer, 1994), but it is not made clear by what mechanism people keep track of previously recalled items to avoid their repetition. Here, WM storage could have a potential role in keeping track of what items have already been reported so that items subsequently sampled from the search set can be checked against to avoid repetitions.

## Current study

To better understand the involvement of WM during episodic retrieval, we introduced a working memory load during retrieval in a free-recall task. While this type of retrieval-specific manipulation has been extensively used in the dual-task literature via the use of a concurrent attention-based task during retrieval (Castel & Craik, 2003; Craik et al., 2010, 1996; Fernandes & Moscovitch, 2000; Naveh-Benjamin et al., 2005, 2000, 2006; Rohrer & Pashler, 2003), the effect of a more storage-directed WM load on episodic retrieval specifically has not been examined.^1^ A WM preload – a four-digit (Experiment 1) or six-digit sequence (Experiment 2) – was shown after each memory list for maintenance during the recall period. Memory for the digit sequence was then tested after the recall window. Similar preload techniques have commonly been used to examine the role of WM across various cognitive domains (Baddeley et al., 2001; Hester & Garavan, 2005; Hinson et al., 2003; Liefooghe et al., 2008; Morey & Cowan, 2004; Sprenger et al., 2011; Turner & Schley, 2016). While the impact of a WM preload on recognition memory (Baddeley et al., 1984) and retrieval from WM (Vergauwe et al., 2014) has been investigated, we are unaware of this manipulation being used to examine the specific involvement of WM during retrieval from episodic memory, nor to examine potential effects on temporal contiguity and recall latencies. Experiment 2 was run as a follow-up to Experiment 1 to confirm that the observed effect on key measures was not due to the maintenance of the four-digit preload insufficiently loading WM.

To assess the relative contribution of the storage aspects of WM to episodic retrieval, a variety of free-recall benchmarks that have been shown to represent fundamental aspects of episodic memory were used. First, we examined how the manipulation of WM load during episodic retrieval influences primacy and recency (a recall advantage for early and late list items, respectively: e.g., Greene, 1986; Murdock, 1962) in both recall accuracy and first recall probabilities (the likelihood of the first item recalled conditionalized on serial position: e.g., Howard & Kahana, 1999). We also considered how WM load potentially influences the degree of temporal contiguity associated with free recall by examining lag-recency via differences in response probabilities conditionalized on the difference in serial positions between successive retrievals or ‘lags’ (lag-CRPs: Kahana, 1996). Lastly, free-recall latencies were modeled using the standard exponential model (Rohrer & Wixted, 1994; Wixted , Rohrer, 1994), to examine potential differences in the rate of memory search during free recall due to the presence of WM load during retrieval. Together, these metrics allowed the examination of the involvement of the storage and attentional aspects of WM during the search and sampling of episodic memory.

Given their similarity in procedure and results, we concurrently report Experiments 1 and 2 below. The preregistration for these experiments is available at https://osf.io/h49w5. The experiments were preregistered via the Open Science Framework (OSF; https://osf.io/), with all preregistration, analysis scripts, and data being made available via the OSF URLs provided alongside the reporting of each experiment. Any deviations from the preregistered analysis plan are noted where necessary.

## Methods

### Participants

Participants (Experiment 1: $$N=53$$; Experiment 2: $$N=52$$) were recruited via Prolific Academic (https://www.prolific.ac/), an online crowdsourcing platform specifically tailored towards recruitment of participants for psychology research, and has been used to successfully replicate several established findings from the psychology literature (Peer et al., 2017). All participants were between 18 and 40 years of age (Experiment 1: $$M = 30.48$$, $$SD = 5.66$$; Experiment 2: $$M = 29.31$$, $$SD = 5.51$$). To ensure a suitable, high-quality sample, recruitment was restricted to participants who met the following eligibility requirements: An approval rating on Prolific Academic of over 90%; and participants with at least five, but no more than 10,000 submissions. All participants were current residents of Australia, New Zealand, the USA, Canada, the UK, or Ireland. Participants who had previously participated in other related studies run by our lab on Prolific Academic were also excluded. Participants who met the eligibility criteria were selected on a first-come, first-served basis and were reimbursed £3 (approximately $4.90 AUD/$3.90 USD) for completing the experiment, which took approximately 30 min.

### Materials

Words for the free-recall task were selected from the EMOTE word database, a database of words pre-rated on various cognitive and socio-emotional dimensions relevant to free recall (Grühn, 2016). All words ($N$ = 172) were 2–3 syllable nouns and were low-medium frequency (KF = 2–290), low on arousal (arousal rating <4 on a 1–7 scale), and were either of positive (valence rating$>4$ on a 1–7 scale) or negative valence (valence rating$<3$). Unique eight-word word lists ($N$=22) were then generated via random sampling-without-replacement from the word pool for each participant, half of which involved sampling five positively valenced and three negative valenced words (resulting in word lists that were on average positively valence), while the other half consisted of five negatively and three positively valenced words (resulting in word lists that were on average negatively valenced).^2^ A list length of eight was chosen to be relatively short to allow collection of data from more trials, but long enough to be sufficiently similar in performance to free recall from longer lists (e.g., Ward et al., 2010).

The digit sequences presented during the experiment were generated per trial by randomly sampling without replacement from the digits 1–9.

### Procedure

Eligible participants were provided with a link to the experiment after signing up for the study. Informed consent was obtained by endorsing an online consent form, after which instructions for each task involved in the experiment were presented. These instructions included a demonstration of each task, with an emphasis on accurately maintaining the digit sequence over recalling the word lists. A short multiple-choice questionnaire was presented at the end of the instruction block to ensure comprehension of experiment instructions. If participants answered all questions correctly, they proceeded to the experiment. Otherwise, they reviewed the instructions again and reattempted the questionnaire.

On each trial, a central fixation cross appeared for 2000 ms. Following a delay of 500 ms, sequential presentation of the word list occurred, with each word being displayed for 1000 ms with a 100-ms inter-stimulus interval. Following the presentation of the last (8th) word in the word list, another central fixation cross (2000 ms) and a delay (500 ms) were presented, which was followed by the sequential presentation of the four-digit sequence. With each digit displayed for 1250 ms, a 100-ms inter-stimulus interval was maintained. Both word lists and digit sequences were presented in 27-point sans-serif font.

How participants interacted with the digit sequence varied based on the experimental condition. In the WM load condition, participants were asked to perform a “digit remembering task”. In this task, participants were to retain the exact four-digit (Experiment 1) or six-digit (Experiment 2) sequence they were shown during the free-recall phase of the current trial. Participants were asked to perform serial recall of the digit sequence after the free-recall phase by typing the digits in their presented order. Participants were given performance-based feedback on the average accuracy of their serial recall of the digit sequences from the last three trials after every third trial. If the average accuracy was below 75%, participants were reminded that they should be trying to perform the digit remembering task as accurately as possible.

In the no-load condition, participants were asked to perform a “digit copying task”, which involved shadowing the digits as they were presented by pressing the number key on the top row of the keyboard that corresponded to the currently displayed digit. The purpose of the digit copying task was to match conditions to the load task in terms of attending to the digits, but without the requirement for maintenance of the digits subsequent to their presentation. No performance-based feedback was given on performance on the digit copying task. To ensure participants understood which version of the digit task they should currently be performing, the name of the task was displayed throughout each trial at the top of the screen. Additionally, a prompt to either “remember these digits” (during the digit load task) or to “copy these digits” (during the digit copying task) accompanied the presentation of each digit sequence.

Following the presentation of the digit sequence, participants had 30 s to recall as many words in any order from the most recently presented word list. Responses were typed into an on-screen text box individually, with participants pressing the enter key after typing each word. The current trial ended once the 30-s response window elapsed, or participants had entered a maximum of eight responses. Participants completed a block of 11 trials under both the WM load and no-load conditions, with the block order being counterbalanced across participants. Upon completion of the experiment, participants were debriefed.

The experiments were written in JavaScript using JsPsych v5.0 (De Leeuw, 2015).

### Data analysis

For inferences regarding the nature of all effects examined, we rely on Bayes factors using the ‘BayesFactor’ package in R (Morey & Rouder, 2018). For all Bayesian ANOVAs, main effects and interaction terms were included as fixed effects, and a random effect of participant on the overall mean was also included. The default priors of the BayesFactor package were used, as they were judged to suitably represent the scale of effects typically seen in cognitive psychology. Bayes factors (BFs) represent the relative evidence for or against a given effect within a model, based on the ratio between the marginal likelihoods associated with the data of a model that includes the effect of interest vs. a null model (which presumes no effect). Specifically, $BF_{10}$ denotes the level of evidence in favor of a given effect and was calculated on the basis of the marginal likelihood for the fully saturated model with all possible main effects and interaction terms included, relative to the marginal likelihood for the full model without the effect in question. $BF_{01}$ is the reciprocal of $BF_{10}$ and represents the evidence in favor of the null model that there is no effect. $BF_{10} > 1$ (which is equivalent to $BF_{01} < 1$) represents evidence in favor an effect, while $BF_{01} > 1$ (and thus $BF_{10} < 1$) is evidence against an effect and in favor of the null model. Based on Kass and Raftery (1995)’s guidelines, we interpreted $BF$>3 to constitute some evidence, while $BF$>10 to be strong evidence.

**Fig. 1:**
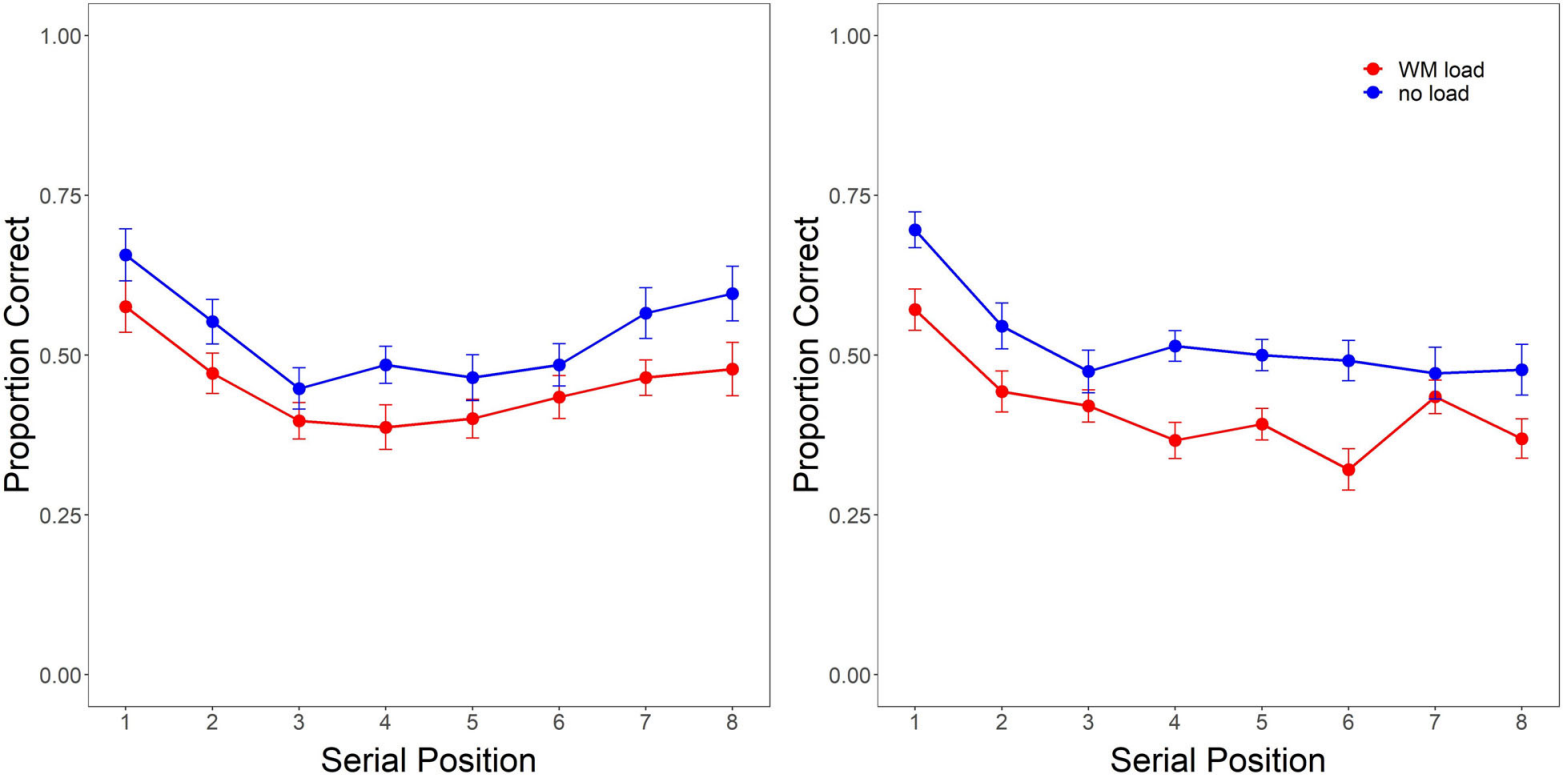
Serial position functions for recall under storage-based WM load vs. no-load from Experiment 1 (four-digit preload, *left*) and 2 (six-digit preload, *right*)

For the free-recall data collected, we scored as correct any typed response (Experiment 1) or transcription of verbal response (Experiment 2) if they were associated with Damerau–Levenshtein distance $<= 1$. Damerau–Levenshtein distance represents the number of character changes via either substitution, deletion, or insertion needed for two strings of characters to be completely identical. Lag-CRPs were calculated as per Kahana (1996).

The ex-Gaussian model was fit to the cumulative response times associated with correct recalls to analyze the rate of memory search. The ex-Gaussian distribution is the convolution of a Gaussian distribution and the exponential distribution and has often been used to model free-recall retrieval latencies (e.g., Rohrer & Pashler, 2003; Unsworth & Engle, 2007; Wixted & Rohrer, 1994). When applying an ex-Gaussian model to retrieval latencies, the Gaussian component – defined by a location parameter, $\mu$, and a scale parameter, $\sigma$ – is used to capture the delay period between the start of the recall window and when the first response is made (which is assumed to be normally distributed: Wixted & Rohrer, 1993). The exponential component is specified by a rate parameter, $\tau$, capturing the exponentially increasing response times during the recall window until participants are unable to retrieve any more list items and recall eventually asymptotes, and is used here to index how impairing WM during retrieval potentially slows down our ability to search memory (e.g., Rohrer & Pashler, 2003). While some previous studies have estimated $N$ in the pure-death model as part of the ex-Gaussian fitting, we relied on the average number of words recalled in each condition as estimates of asymptotic recall.

The ex-Gaussian models of response time distributions for each experiment were fit using hierarchical Bayesian modeling using the BRMS package for R (Bürkner, 2017). Intercept-only models were used to fit an ex-Gaussian distribution to cumulative response times for correct recalls; the distribution was truncated to exclude response times outside the range 0—30 s for Experiment 1. We used the default specifications for fitting the ex-Gaussian family of distributions of the BRMS package, which we considered to be reasonable default priors for this application.^3^ The default model link function of BRMS for fitting ex-Gaussian distributions is an identity link with a log link function for estimating the $\sigma$ and $\beta$ (equivalent to $\tau$) parameters. The default value used for the adaptive delta parameter was .8. We used four Markov chain Monte Carlo (MCMC) chains with 11,000 iterations each to estimate the posterior distributions for each parameter of the ex-Gaussian models, discarding the first 10,000 iterations from each chain as a ‘burn-in’ period. Estimates for all parameters reported were associated with $R-hat$ = 1, indicating convergence of all four MCMC chains.

## Results

Participants with poor performance on either digit task (<80% accuracy for Experiment 1; <50% accuracy for Experiment 2), recalled less than one word on average on the free-recall task per trial, or did not complete at least 80% of trials were removed prior to analysis leaving a final $N$ of 27 (Experiment 1) and 32 (Experiment 2).^4^ Digit task accuracy for the final samples was $M(SD) = .90(.06)$ for the digit copying task and $M(SD) = .92(.06)$ for the digit remembering task (Experiment 1), and $M(SD) = .92(.09)$ for the digit copying task and $M(SD) = .82(.13)$ for the digit remembering task (Experiment 2).

### Free-recall benchmarks

To examine how working memory load influenced the typical primacy and recency associated with free recall, Fig. 1 shows recall accuracy as a function of serial position for retrieval under load vs. no-load. Results of a 2 (WM load vs. no-load) $\times$ 8 (serial position) Bayesian ANOVA of recall accuracy for each experiment revealed strong evidence in favor of an effect of load when using both a four-digit (Experiment 1: $BF_{10} = 1.95e03$) and six-digit preload (Experiment 2: $BF_{10} = 2.89e08$), with retrieval under a storage-based WM load being associated with a 15–20% drop in overall recall accuracy. We also found strong evidence in favor of an effect of serial position for both experiments (Experiment 1: $BF_{10} = 1.95e03$; Experiment 2: $BF_{10} = 2.89e08$), consistent with the recency shown in Fig. 1 for both conditions in Experiment 1 and the overall primacy observed for both condition across experiments. However, the presence of a WM load during retrieval did not appear to influence the relative degree of primacy or recency, as we found strong evidence against any interaction between load and serial position during free recall under either a four- or six-digit load (Experiment 1: $BF_{01} = 114.81$; Experiment 2: $BF_{01} = 29.66$).

**Fig. 2:**
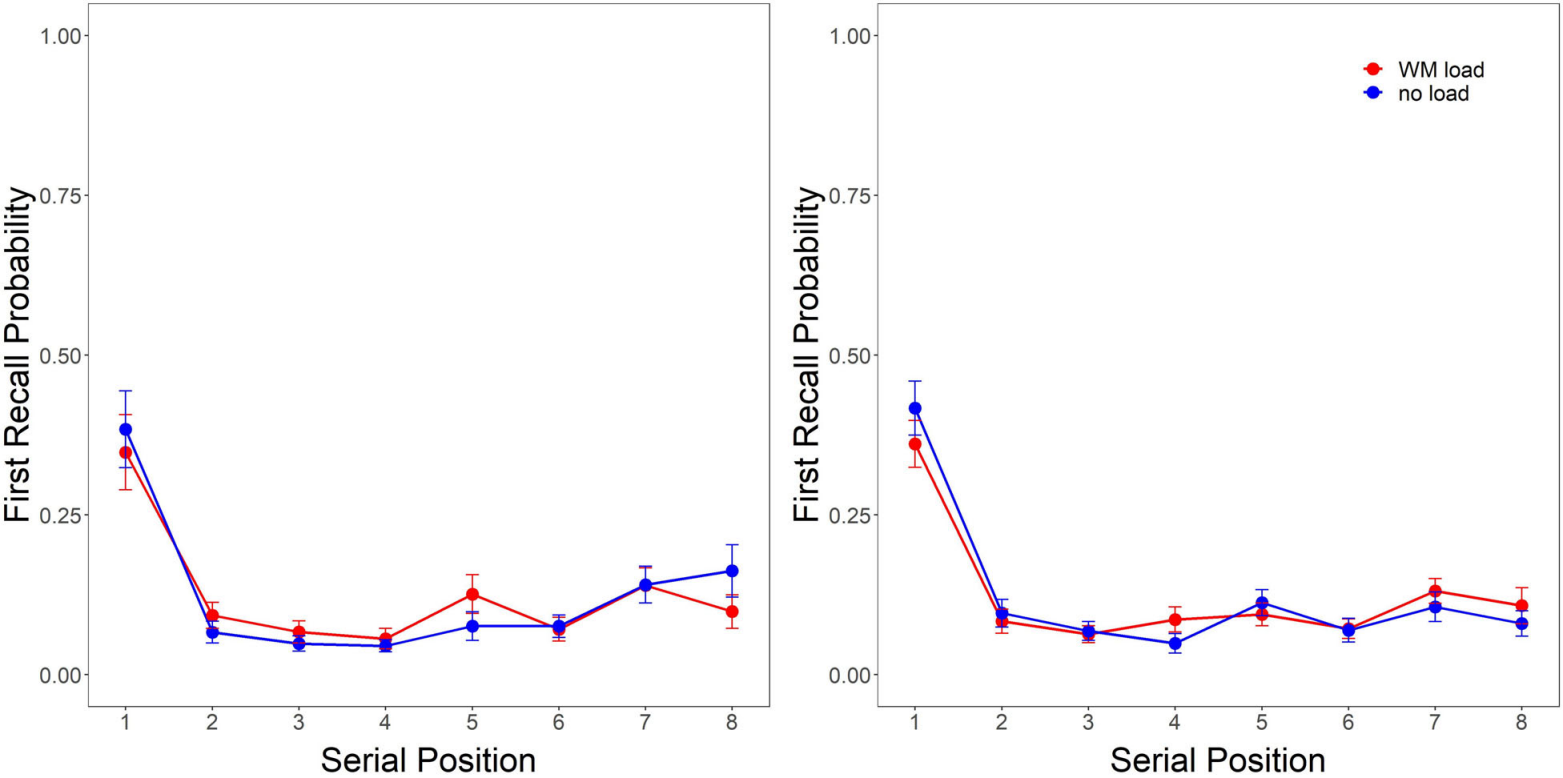
First recall probability functions for recall under storage-based WM load vs. no-load from Experiment 1 (four-digit preload, *left*) and 2 (six-digit preload, *right*)

First recall probabilities (FRP) for both experiments are presented in Fig. 2. A 2 (WM load vs. no-load) $\times$ 8 (serial position) Bayesian ANOVA provided support for the consistent primacy observed for both conditions across experiments, with consistent strong evidence in favor of an effect of serial position (Experiment 1: $BF_{10} = 2.51e25$; Experiment 2: $BF_{10} = 1.25e49$). However, we observed no effect of WM load influencing FRPs, finding strong evidence against both an effect of load (Experiment 1: $BF_{01} = 8.87$; Experiment 2: $BF_{01} = 10.07$) and a load $\times$ serial position interaction (Experiment 1: $BF_{01} = 34.99$; Experiment 2: $BF_{01} = 31.96$).

Finally, we examined how WM load influenced the output order of list items during free recall by examining conditional response probabilities as a function of lag (lag-CRP). Lags represent the difference between the serial position of the last and subsequently recalled list item; with smaller lags representing subsequent recalls coming from nearby serial positions and larger lags represent subsequent recall from distal serial positions. The sign of the lag denotes whether the subsequent recall proceeded in a forward (positive) or backward (negative) direction along the word list.

**Fig. 3:**
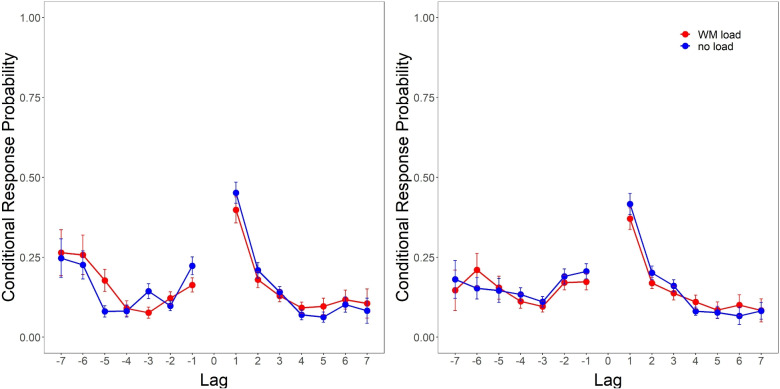
Conditional response probability as a function of recall lag under storage-based WM load vs. no-load from Experiment 1 (four-digit preload, *left*) and 2 (six-digit preload, *right*)

Figure 3 shows CRPs for free recall under WM load vs. no-load. A 2 (WM load vs no-load) $\times$ 8 (lag -4 to +4)^5^ Bayesian ANOVA of lag-CRPs found strong evidence in favor of an effect of lag, supporting the observed overall preference for +1 lags (Experiment 1: $BF_{10} = 2.11e41$; Experiment 2: $BF_{10} = 2.82e22$). However, WM load did not appear to have any substantial impact on lag-recency in either experiment, with non-trivial evidence against an effect of load (Experiment 1: $BF_{01} = 2.96$; Experiment 2: $BF_{01} = 7.80$), and strong evidence against a load $\times$ lag interaction (Experiment 1: $BF_{01} = 147.16$; Experiment 2: $BF_{01} = 207.44$).

To assess the potential role of the storage component of WM in keeping track of recalled items so as to avoid erroneous repetitions during free recall, we compared the frequency of repetitions made under WM load vs. no load (versus all other response types) using a chi-squared analysis. Because there were so few repetitions per participant, this analysis was performed on the frequencies aggregated across participants. Results of this analysis revealed no effect of WM load on the frequency of repetitions during either a four-digit or six-digit WM load (Experiment 1: $\chi ^2$(1)=0.75, $p$ =.38; Experiment 2: $\chi ^2$(1)=.001, $p$ =.96).

**Fig. 4:**
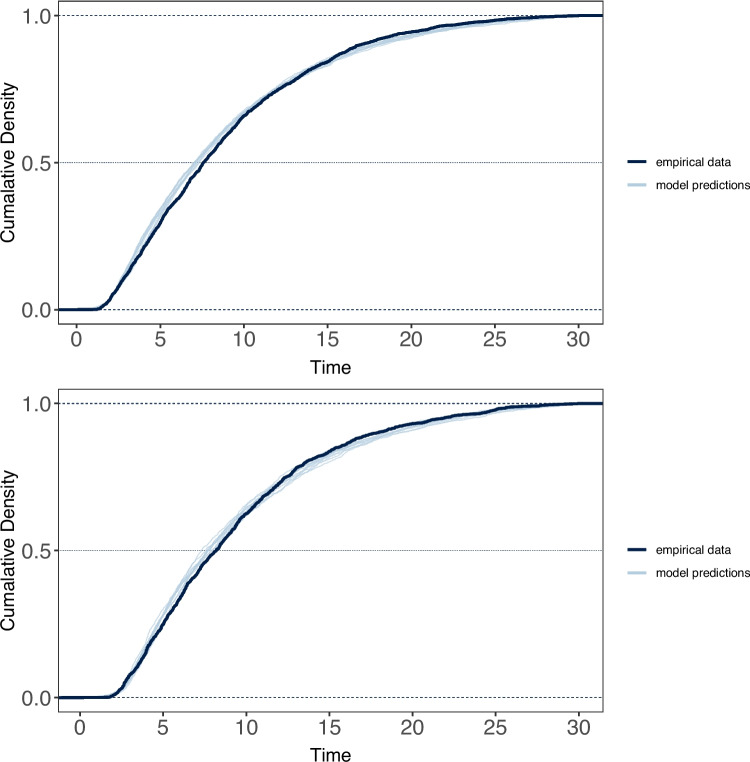
Cumulative density functions for free-recall latencies under WM load (*top*) and no WM load (*bottom*) for Experiment 1 (four-digit WM preload), overlaid with posterior samples from the estimated ex-Gaussian models

### Recall latency modeling

**Fig. 5:**
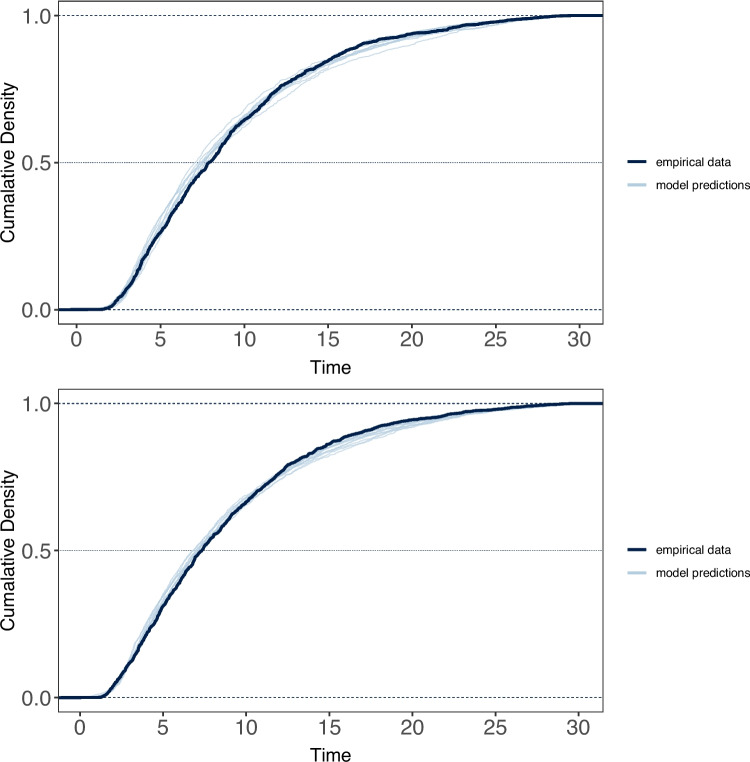
Cumulative density functions for free-recall latencies under WM load (*top*) and no WM load (*bottom*) for Experiment 2 (six-digit WM preload), overlaid with posterior samples from the estimated ex-Gaussian models

To examine the effect of the load on the dynamics of retrieval, we fit the cumulative ex-Gaussian model to cumulative response time distributions associated with correct recalls. Figures 4 and 5 show the cumulative distribution functions (CDFs) associated with correct recalls under load and no-load for Experiments 1 and 2, overlaid with posterior samples from the estimated ex-Gaussian models used to give some indication of the goodness of fit.

However, there is a consistent small overestimation of recall latencies early in the recall period, and underestimation for later recalls. This pattern of results is consistent with earlier studies of free-recall response times that found similar patterns when fitting ex-Gaussian models, arguing that the pattern of misfitting likely represents the impact of temporal clustering of responses at different periods during recall (Rohrer & Wixted, 1994).

The means of the posterior estimates associated with the parent distributions of the parameters for the ex-Gaussian model of cumulative recall latencies are presented in Table 1. Comparisons of parameter estimates between the WM load vs. no-load conditions based on the associated 95% credible intervals suggest that for both experiments, estimates for $\mu$ were noticeably higher for recall under load, which is consistent with the longer delay before recall onset that was observed in the respective cumulative RT distributions. Critically, the lack of any substantial difference in estimates for $\tau$ across conditions for both experiments supports the apparent lack of difference in the rate of approach to asymptotic recall observed in the cumulative RT distributions for both experiments, suggesting that the presence of a storage-based WM load did not appear to meaningfully slow the rate of episodic retrieval relative to recall under no load. There were also no substantial differences in estimates for $\sigma$ between conditions.

**Table 1:** Means (95 $\%$ credible intervals) for the posterior distributions associated with each parameter of the ex-Gaussian model of average cumulative response times for recall under a storage-based WM load vs. no-load for Experiments 1 (four-digit preload) and 2 (six-digit preload)

	Experiment 1		Experiment 2	
Parameters	No-load	Load	No-load	Load
$\mu$	1.94 (1.75, 2.15)	2.55 (2.34, 2.78)	2.04 (1.82, 2.27)	2.60 (2.40, 2.82)
$\sigma$	0.58 (0.41,0.77)	0.66 (.46, .88)	0.67 (0.45,0.91)	0.72 (0.54, 0.94)
$\tau$	7.79 (7.28, 8.35)	7.75 (7.17, 8.37)	7.39 (6.84, 7.94)	7.31 (6.75, 7.93)

An interesting peripheral result of this was the modest increase in $\mu$ for retrieval under preload, which captures the typically observed delay of recall onset at the start of the recall. Previous applications of the ex-Gaussian to recall data have found that increasing the retention interval from 3 to 27 s increased $\mu$, arguing that this Gaussian component represents the time taken to establish the search set prior to recall onset (Wixted , Rohrer, 1993).

While the presence of a storage-based load in WM at retrieval may have increased the time taken to establish the search set due to having to switch between the maintenance of the digit sequence and generating the retrieval cue used to establish the search set, the observed shift in $\mu$ could also be potentially explained by participants simply rehearsing (e.g., Baddeley & Hitch, 1974) or refreshing the digit sequence in WM (e.g., Camos et al., 2009 prior to starting recall for the word list.

The results from the latency analysis are also compelling in ruling out a potential interaction effect. As recall progresses, we might expect to see recall slow down, either because the demands on WM increase, such as keeping track of those items already recalled, or because search itself becomes more difficult and thus more reliant on WM to continue effective retrieval. Such an effect was observed by Unsworth and Engle (2007) in individual differences in WM, whereby higher and lower WMC people had similar retrieval rates earlier in recall, but a more pronounced slowing in lower WMC participants as recalled progressed. An apparent lack of effect of load on the shape or rate appears to rule out such a possibility here.

## Discussion

In this study, we investigated the potential role of working memory storage capacity, particularly during episodic retrieval. To this end, we imposed a storage-based WM load during retrieval in a free-recall task by requiring participants to maintain and report a random digit sequence presented after presentation of the to-be-remembered items but prior to the start of the recall period. While the WM preload manipulation produced a 15–20% drop in overall recall accuracy compared to a control, there was no effect on other benchmark effects of free recall examined. Specifically, we observed no effect of WM load on primacy/recency effects during recall (Greene, 1986; Murdock, 1962) and recall initiation (Howard & Kahana, 1999), lag-recency (Kahana, 1996), suggesting that WM load did not systematically influence the relative accessibility of individual list items during retrieval but impaired access to all items. Further, we did not observe an effect of WM load on the rate of memory search via modeling of recall latencies, suggesting that the digit remembering task did not substantially divert attention away from retrieval (Naveh-Benjamin et al., 2006; Rohrer & Pashler, 2003), or influence the size of the search in memory (Unsworth & Engle, 2007).

The overall recall accuracy under a storage-based WM load at retrieval we observed was somewhat smaller than the ~25% decrease reported in previous dual-studies that used a difficult attention-based concurrent task with incongruent stimulus-response mappings (Naveh-Benjamin et al., 2006; Rohrer & Pashler, 2003). While the more negligible effect on recall accuracy and the lack of change in the rate of memory search compared to previous dual-task studies could suggest that a storage-based WM load is less detrimental to episodic retrieval than an attention-based WM load, an alternative explanation for this lack of effect is that the storage-based nature of the concurrent task used during retrieval (i.e., maintaining the digit sequence in memory) was not sufficiently attention-demanding to force attention away from retrieval, thus allowing for both retrieval and digit maintenance occurring effectively in parallel.

If attending to the primary recall and concurrent preload tasks in parallel was possible, why then did we observe a drop in recall accuracy? One explanation that is consistent with both the lack of effect on accessibility and rate of memory search is that the WM load introduced additional interference at the point of retrieval, which made some items unrecoverable. That is, from the perspective of sampling-based models, load had no effect on the probability of sampling of traces, and specifically lowered the probability with which a sampled trace was converted into an overt report (cf. Raaijmakers & Shiffrin, 1980; Unsworth, 2015; Wixted et al., 1997). This is consistent with simulation work by Unsworth (2007) examining how individual differences in WM influence episodic retrieval; Unsworth (2007) demonstrated that one way that lower WM capacity individuals could potentially be associated with lower recall accuracy without a change in recall latencies (thus search set size) is that some target items are unrecoverable due to poor encoding.

Given that the design of our study necessarily left encoding intact, this unrecoverability could potentially reflect the presence of WM load disrupting the ability to recover enough information during retrieval to sufficiently activate items on a given trial (e.g., Raaijmakers & Shiffrin, 1980; Wixted et al., 1997). For example, Farrell et al. (2012) assumed that for an item to be retrieved, its normalized activation level (modified by noise) needed to exceed a recoverability threshold. The effect of WM load on recall accuracy without impairing lag-recency could reflect a reduction in the potential activation of list items. The results are analogous to those of Rohrer and Wixted (1994) and Wixted et al. (1997), who found that making items “stronger’ ’ by giving them more or longer presentations at study, affected only the probability of recall (and not recall latency) when all items on a list were strengthened. One mechanism for this, suggested by a reviewer, is that the digits – being very different from the words – steal activation from the words and thus make them less recoverable, and the relationship between the list items and the load may impact on recoverability. Given that trials were blocked by condition, it is also possible that participants in the load condition encoded the list items less deeply to avoid strengthening them to the extent that they interfered with the digits task.

Equally, how is it that participants were well able to retrieve the load – showing its effective maintenance – without impacting on recall characteristics other than overall recall probability? One explanation is that retrieval from episodic memory is not demanding on WM storage. One potential role for WM in episodic retrieval, as outlined in the Introduction, was in keeping track of recalled items to avoid repetition errors. This would predict that loading WM would then leave less capacity for remembering which items were recalled in the free-recall task. Load did not affect the frequency of repetition errors in the free-recall task. Instead, this provides some evidence for repetition errors in free recall being minimized by suppression of recalled items (e.g., Farrell, 2012) or by the use of recalled items to cue other list items (e.g., Howard & Kahana, 2002). More generally, the implication is that the retrieval operations in models such as TCM (Howard & Kahana, 2002), CRM (Polyn et al., 2009), and SAM (Raaijmakers & Shiffrin, 1980) are not dependent on storage, and may relate more to attentional aspects of working memory.

Another non-exclusive explanation is that it is not meaningful to discuss a separate working memory storage capacity; instead, consider unitary models for working and episodic memory (e.g., Brown et al., 2007; Farrell, 2012). Farrell et al. (2012) showed that his model could account for short-term and long-term memory, including correlations between working memory and episodic memory performance (e.g., Unsworth et al., 2010) by assuming that continuous experiences are partitioned into groups, and that short-term or working memory is simply the group whose temporal context is currently active. Farrell et al. (2012) accounted for delayed recall by assuming that the distractors following a list are placed in their own group to protect the list. This account could be extended to the current results by assuming that the digits are placed in their own group, and can be recalled after the free-recall period by targeted retrieval of that group.

The results of our study have also raised some novel implications regarding both extant dual-task and individual differences in WM literatures regarding the involvement of WM during episodic retrieval. Firstly, our observed drop in recall accuracy without any substantive change in the rate of memory search under WM load suggests that these two effects can be dissociated. In the dual-task and WM literatures, it has tended to be the case that factors that affect recall accuracy are at least partly due to changes in search, whereas we have shown that recall accuracy can result from changes in recoverability. Secondly, while others have argued that non-recoverability of items from episodic memory can arise from encoding deficiencies (e.g., Tulving & Pearlstone, 1966; Unsworth, 2007; 2008), we have demonstrated that similar effects on recoverability can potentially arise at the point of retrieval.

Overall, this research has demonstrated that a largely storage-based WM load, while influencing overall recall accuracy, does not appear to fundamentally influence how people search episodic memory during retrieval. Overall, this argues against a unique role of the purely storage aspects of WM during episodic retrieval. However, the results of this study have also raised some interesting questions regarding the relationship between search set size effects seen in the WM literature and the differences in the rate of memory search from dual-task studies. Finally, an impairment of episodic retrieval without an apparent change in temporal contiguity also suggests the need for further modeling of effects on the recoverability of items in memory at the point of retrieval, as recoverability effects have only been considered from the point of encoding by contemporary models of episodic memory. Together, these results set interesting challenges for future studies in both episodic and working memory literature to meet in order to gain a more complete understanding of the relationship between WM and episodic retrieval.

## Figures and Tables

**Fig. 6:**
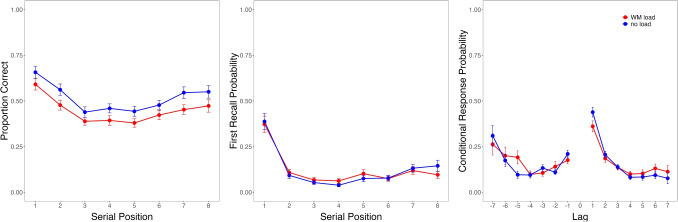
Serial recall, first recall, and conditional lag-probability functions for Experiment 1 when including participants who otherwise were excluded based on performance on the digit copying task

**Fig. 7:**
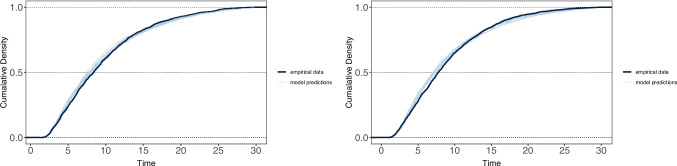
Cumulative recall times for Experiment 1 when including participants who otherwise were excluded based on performance on the digit copying task

## Data Availability

This work was preregistered on the Open Science Framework, with all data, study materials, and analysis code being made available at https://osf.io/h49w5.

## Appendix A Within-subjects ANOVA table

**Table 2:** Within-subjects ANOVA results for serial position, first recall and conditional response probability-lag functions in Experiments 1 (four-digit preload) and 2 (six-digit preload)

Experiment	Dependent variable	Effect	*F* test
1	recall accuracy	SP	*F*(7, 217) = 9.77, *p* ** = 1.81e-06**
		WM load	*F*(1, 31) = 37.98,*p* = 7.72e-07
		SP x WM load	*F*(7, 217) = 1.08, *p* = 0.37
	first recall probability	SP	*F*(7, 259) = 20.79, *p* ** = 3.11e-10**
		WM load	*F*(1, 37) = -3.92e-14, *p* = 0.99
		SP x WM load	*F*(7, 259) = 1.52, *p* ** = 0.21**
	conditional response probability (lag)	lag	*F*(7, 259) = 42.90, *p* = 1.09e-22
		WM load	*F*(1, 37) = 13.01, *p* = 9.09e-04
		lag x load	*F*(7, 259) = 1.50, *p* = 0.17
2	recall accuracy	SP	*F*(7, 217 = 9.77, *p* ** = 1.81e-06**
		WM load	*F*(1, 31) = 37.98, *p* = 7.72e-07
		SP x load	*F*(7, 217) = 1.08, *p* ** = 0.37**
	first recall probability	SP	*F*(7, 217) = 3.47, *p* ** = 8.92e-16**
		WM load	*F*(1, 31) = 2.75e-15, *p* = .99
		SP x load	*F*(7, 217) = 1.03, *p* ** = 0.39**
	conditional response probability (lag)	lag	*F*(7, 217) = 33.38, *p* ** = 3.73e-19**
		WM load	*F*(1, 31) = 4.83,*p* = 3.55e-02
		lag x load	*F*(7, 217)= 0.28, *p* = 9.59e-01

Note. SP - Serial position; WM - Working memory; *p* values in bold have been Greenhouse–Geisser corrected due to violations of sphericity (i.e., Mauchly’s test was significant for these effects)

## Appendix B Analysis of Experiment 1 with lax exclusion criteria

Figures 6 and 7 show the average performance when including participants who otherwise were excluded based on performance on the digit copying task. The pattern shown here is very similar to that in the main experiment, with an effect of load only on overall recall accuracy.

## Footnotes

1 While Baddeley et al. (1984) examined the effect of a digit-based concurrent task during free recall (Experiment 3), this task was completed during both study and test phases of their experiment and thus confounded any potential effects at retrieval with those at encoding. Further, their task required constant responses during encoding/recall, which places this task closer to the attention-based tasks used in the dual task literature than a storage-focused manipulation.

2 Word lists were constructed based on valence as later experiments in our lab used the same stimuli and similar procedures, but also included an evaluation component.

3 As per the BRMS documentation, the default prior for population-level effects (including monotonic and category-specific effects) is an improper flat prior over the reals. In contrast, standard deviations of group-level random effects were based on a half student-t prior with 3 degrees of freedom and a scale parameter that depends on the standard deviation of the response after applying the link function.

4 The major source of sample attrition in Experiment 1 was 19 participants failing to meet the minimum accuracy for digit copying task as responses associated with copying each digit were recorded sequentially, leading to situations where when the correct response to the current digit was made outside the response window and was counted as an incorrect response to the following digit. While this issue was addressed in Experiment 2, and only seven participants failed to meet the minimum accuracy threshold for the digit copying task, an increased proportion of participants failed to meet the minimum accuracy requirement for the six-digit remembering task. Further, one additional participant in Experiment 1 was removed, as they displayed near-perfect serial recall of all presented eight-word lists. This fact, in combination with the near-linear response times they produced during recall and the unsupervised nature of testing online was taken to indicate that they may have been recording the word during list presentation. Appendix B shows that the results are very similar when the exclusion criteria are relaxed.

5 While including all possible lags in the lag-CRP functions, we restricted the analysis of lag-CRP estimates to this range as larger lags tend to be relatively rare and thus lead to unstable estimates of associated response probabilities

## References

[CR1] Atkinson, R. C., & Shiffrin, R. M. (1968). Human memory: A proposed system and its control processes. In K. W. Spence & J. T. Spence (Eds.), *The Psychology of Learning and Motivation* (Vol. 2, pp. 89–195). New York: Academic Press.

[CR2] Atkinson, R. C., & Shiffrin, R. M. (1971). The control of short-term memory. *Scientific American,**225*, 82–90. 10.1038/scientificamerican0871-82 5089457

[CR3] Baddeley, A., Chincotta, D., & Adlam, A. (2001). Working memory and the control of action: Evidence from task switching. *Journal of Experimental Psychology: General,**130*(4), 641–657. 10.1037/0096-3445.130.4.641 11757873

[CR4] Baddeley, A., & Hitch, G. (1974). Working memory. *Psychology of Learning and Motivation,**8*, 47–89.

[CR5] Baddeley, A., Lewis, V., Eldridge, M., & Thomson, N. (1984). Attention and retrieval from long-term memory. *Journal of Experimental Psychology: General,**113*(4), 518.

[CR6] Bousfield, W. A., Sedgewick, C. H. W., & Cohen, B. H. (1954). Certain Temporal Characteristics of the Recall of Verbal Associates. *The American Journal of Psychology,**67*(1), 111. 10.2307/1418075 13138773

[CR7] Brewer, G. A., & Unsworth, N. (2012). Individual differences in the effects of retrieval from long-term memory. *Journal of Memory and Language,**66*(3), 407–415. 10.1016/j.jml.2011.12.009

[CR8] Brown, G. D. A., Neath, I., & Chater, N. (2007). A temporal ratio model of memory. *Psychological Review,**114*(3), 539–576. 10.1037/0033-295X.114.3.539 17638496 10.1037/0033-295X.114.3.539

[CR9] Bürkner, P.-C. (2017). Brms: An r package for bayesian multilevel models using stan. *Journal of Statistical Software,**80*(1), 1–28.

[CR10] Camos, V., Lagner, P., & Barrouillet, P. (2009). Two maintenance mechanisms of verbal information in working memory. *Journal of Memory and Language,**61*(3), 457–469. 10.1016/j.jml.2009.06.002

[CR11] Castel, A. D., & Craik, F. I. M. (2003). The Effects of Aging and Divided Attention on Memory for Item and Associative Information. *Psychology and Aging,**18*(4), 873–885. 10.1037/0882-7974.18.4.873 14692872 10.1037/0882-7974.18.4.873

[CR12] Chow, M., & Conway, A. R. A. (2015). The scope and control of attention: Sources of variance in working memory capacity. *Memory & Cognition,**43*(3), 325–339. 10.3758/s13421-014-0496-9 25604642 10.3758/s13421-014-0496-9

[CR13] Cowan, N. (1999). An embedded-processes model of working memory. *Models of Working Memory: Mechanisms of Active Maintenance and Executive Control,**20*, 506.

[CR14] Cowan, N. (2017). The many faces of working memory and short-term storage. *Psychonomic Bulletin & Review,**24*(4), 1158–1170. 10.3758/s13423-016-1191-6 27896630 10.3758/s13423-016-1191-6

[CR15] Craik, F. I. M., Luo, L., & Sakuta, Y. (2010). Effects of aging and divided attention on memory for items and their contexts. *Psychology and Aging,**25*(4), 968–979. 10.1037/a0020276 20973605 10.1037/a0020276

[CR16] Craik, F. I. M., Naveh-Benjamin, M., Govoni, R., & Anderson, N. D. (1996). The Effects of Divided Attention on Encoding and Retrieval Processes in Human Memory. *Journal of Experimental Psychology: General,**125*(2), 159–180. 10.1037/0096-3445.125.2.159 8683192 10.1037//0096-3445.125.2.159

[CR17] Daneman, M., & Carpenter, P. A. (1980). Individual differences in working memory and reading. *Journal of Verbal Learning and Verbal Behavior,**19*(4), 450–466. 10.1016/S0022-5371(80)90312-6

[CR18] De Leeuw, J. R. (2015). jsPsych: A JavaScript library for creating behavioral experiments in a web browser. *Behavior Research Methods,**47*(1), 1–12. 24683129 10.3758/s13428-014-0458-y

[CR19] Farrell, S. (2012). Temporal clustering and sequencing in short-term memory and episodic memory. *Psychological Review,**119*(2), 223–271. 10.1037/a0027371 22506678 10.1037/a0027371

[CR20] Fernandes, M. A., & Moscovitch, M. (2000). Divided attention and memory: Evidence of substantial interference effects at retrieval and encoding. *Journal of Experimental Psychology: General,**129*(2), 155–176. 10.1037/0096-3445.129.2.155 10868332 10.1037//0096-3445.129.2.155

[CR21] Greene, R. L. (1986). Sources of Recency Effects in Free Recall. *Psychological Bulletin,**99*(2), 221–228. 10.1037/0033-2909.99.2.221

[CR22] Grühn, D. (2016). *An English Word Database of EMOtional TErms (EMOTE).*10.1177/0033294116658474 10.1177/0033294116658474 27401069

[CR23] Hester, R., & Garavan, H. (2005). Working memory and executive function: The influence of content and load on the control of attention. *Memory and Cognition,**33*(2), 221–233. 10.3758/BF03195311 16028577 10.3758/bf03195311

[CR24] Hinson, J. M., Jameson, T. L., & Whitney, P. (2003). Impulsive decision making and working memory. *Journal of Experimental Psychology: Learning, Memory, and Cognition,**29*(2), 298–306. 10.1037/0278-7393.29.2.298 12696817 10.1037/0278-7393.29.2.298

[CR25] Howard, M. W., & Kahana, M. J. (1999). Contextual variability and serial position effects in free recall. *Journal of Experimental Psychology. Learning, Memory, and Cognition,**25*(4), 923–941. 10439501 10.1037//0278-7393.25.4.923

[CR26] Howard, M. W., & Kahana, M. J. (2002). A distributed representation of temporal context. *Journal of Mathematical Psychology,**46*(3), 269–299. 10.1006/jmps.2001.1388

[CR27] Kahana, M. J. (1996). Associative retrieval processes in free recall. *Memory and Cognition,**24*(1), 103–109. 10.3758/bf03197276 8822162 10.3758/bf03197276

[CR28] Kane, M. J., & Engle, R. W. (2000). Working-memory capacity, proactive interference, and divided attention: Limits on long-term memory retrieval. *Journal of Experimental Psychology. Learning, Memory, and Cognition,**26*(2), 336–358. 10.10371/0278-7393.26.2.336 10764100 10.1037//0278-7393.26.2.336

[CR29] Kass, R. E., & Raftery, A. E. (1995). Bayes Factors. *Journal of the American Statistical Association,**90*(430), 773–795. 10.1080/01621459.1995.10476572

[CR30] Liefooghe, B., Barrouillet, P., Vandierendonck, A., & Camos, V. (2008). Working memory costs of task switching. *Journal of Experimental Psychology: Learning, Memory, and Cognition,**34*(3), 478–494. 10.1037/0278-7393.34.3.478 18444750 10.1037/0278-7393.34.3.478

[CR31] Lohnas, L. J., Polyn, S. M., & Kahana, M. J. (2015). Expanding the scope of memory search: Modeling intralist and interlist effects in free recall. *Psychological Review,**122*(2), 337–363. 10.1037/a0039036 25844876 10.1037/a0039036

[CR32] McGill, W. J. (1963). Stochastic latency mechanisms. In R. D. Luce, R. R. Bush, & E. Galanter (Eds.), *Handbook of mathematical psychology* (Vol. 1, pp. 309–360). New York: Wiley.

[CR33] Mogle, J. A., Lovett, B. J., Stawski, R. S., & Sliwinski, M. J. (2008). What’s so special about working memory? An examination of the relationships among working memory, secondary memory, and fluid intelligence. *Psychological Science,**19*(11), 1071–1077. 19076475 10.1111/j.1467-9280.2008.02202.x

[CR34] Morey, & Cowan, N. (2004). When visual and verbal memories compete: Evidence of cross-domain limits in working memory. *Psychological Bulletin & Review,**11*(2), 296–301. 10.3758/bf03196573 15260196

[CR35] Morey, & Rouder, J. N. (2018). *BayesFactor: Computation of bayes factors for common designs*. https://CRAN.R-project.org/package=BayesFactor

[CR36] Murdock, B. B. (1962). The serial position effect of free recall. *Journal of Experimental Psychology*, *64*(5), 482–488. 10.1037/h0045106

[CR37] Naveh-Benjamin, M., Craik, F. I. M., Guez, J., & Dori, H. (1998). *Effects of divided attention on encoding and retrieval processes in human memory: Further support for an asymmetry*. 10.1037/0278-7393.24.5.1091 10.1037//0278-7393.24.5.1091 9747524

[CR38] Naveh-Benjamin, M., Craik, F. I. M., Guez, J., & Kreuger, S. (2005). Divided attention in younger and older adults: effects of strategy and relatedness on memory performance and secondary task costs. *Journal of Experimental Psychology. Learning, Memory, and Cognition,**31*(3), 520–537. 10.1037/0278-7393.31.3.520 15910135 10.1037/0278-7393.31.3.520

[CR39] Naveh-Benjamin, M., Craik, F. I. M., Perretta, J. G., & Tonev, S. T. (2000). The effects of divided attention on encoding and retrieval processes: The resiliency of retrieval processes. *The Quarterly Journal of Experimental Psychology Section A,**53*(3), 609–625. 10.1080/713755914 10.1080/713755914 10994220

[CR40] Naveh-Benjamin, M., Kilb, A., & Fisher, T. (2006). Concurrent task effects on memory encoding and retrieval: further support for an asymmetry. *Memory & Cognition,**34*, 90–101. 10.3758/BF03193389 16686109 10.3758/bf03193389

[CR41] Oberauer, K. (2002). Access to information in working memory: exploring the focus of attention. *Journal of Experimental Psychology. Learning, Memory, and Cognition,**28*(3), 411–421. 10.1037/0278-7393.28.3.411 12018494

[CR42] Peer, E., Brandimarte, L., Samat, S., & Acquisti, A. (2017). Beyond the Turk: Alternative platforms for crowdsourcing behavioral research. *Journal of Experimental Social Psychology,**70*, 153–163. 10.1016/j.jesp.2017.01.006

[CR43] Polyn, S. M., Norman, K. A., & Kahana, M. J. (2009). A context maintenance and retrieval model of organizational processes in free recall. *Psychological Review,**116*(1), 129–156. 10.1037/a0014420 19159151 10.1037/a0014420 PMC2630591

[CR44] Raaijmakers, J. G., & Shiffrin, R. M. (1980). SAM: A theory of probabilistic search of associative memoryc. In *The psychology of learning and motivation*.

[CR45] Raaijmakers, J. G., & Shiffrin, R. M. (1981). Search of associative memory. *Psychological Review,**88*(2), 93–134. 10.1037/0033-295X.88.2.93

[CR46] Rohrer, D., & Pashler, H. E. (2003). Concurrent task effects on memory retrieval. *Psychonomic Bulletin & Review,**10*(1), 96–103. 10.3758/BF03196472 12747495 10.3758/bf03196472

[CR47] Rohrer, D., & Wixted, J. T. (1994). An analysis of latency and interresponse time in free recall. *Memory & Cognition,**22*(5), 511–524. 10.3758/BF03198390 7968547 10.3758/bf03198390

[CR48] Sederberg, P. B., Howard, M. W., & Kahana, M. J. (2008). A context-based theory of recency and contiguity in free recall. *Psychological Review,**115*(4), 893–912. 10.1037/a0013396 18954208 10.1037/a0013396 PMC2585999

[CR49] Shipstead, Z., Lindsey, D. R. B., Marshall, R. L., & Engle, R. W. (2014). The mechanisms of working memory capacity: Primary memory, secondary memory, and attention control. *Journal of Memory and Language,**72*, 116–141. 10.1016/j.jml.2014.01.004

[CR50] Shipstead, Z., Redick, T. S., Hicks, K. L., & Engle, R. W. (2012). *The scope and control of attention as separate aspects of working memory.*10.1080/09658211.2012.691519 22734653 10.1080/09658211.2012.691519

[CR51] Spillers, G. J., & Unsworth, N. (2011). Variation in working memory capacity and temporal-contextual retrieval from episodic memory. *Journal of Experimental Psychology: Learning, Memory, and Cognition,**37*(6), 1532–1539. 10.1037/a0024852 21823812 10.1037/a0024852

[CR52] Sprenger, A. M., Dougherty, M., Atkins, S. M., Franco-Watkins, A. M., Thomas, R., Lange, N., & Abbs, B. (2011). Implications of cognitive load for hypothesis generation and probability judgment. *Frontiers in Psychology,**2*, 129. 21734897 10.3389/fpsyg.2011.00129 PMC3120978

[CR53] Tulving, E. (1983). *Elements of Episodic Memory* (Vol. 2).

[CR54] Tulving, E., & Pearlstone, Z. (1966). Availability versus accessibility of information in memory for words. *Journal of Verbal Learning and Verbal Behavior,**5*(4), 381–391. 10.1016/S0022-5371(66)80048-8

[CR55] Turner, B. M., & Schley, D. R. (2016). The anchor integration model: A descriptive model of anchoring effects. *Cognitive Psychology,**90*, 1–47. 10.1016/j.cogpsych.2016.07.003 27567237 10.1016/j.cogpsych.2016.07.003

[CR56] Unsworth, N. (2007). Individual differences in working memory capacity and episodic retrieval: examining the dynamics of delayed and continuous distractor free recall. *Journal of Experimental Psychology: Learning, Memory, and Cognition,**33*(6), 1020–1034. 10.1037/0278-7393.33.6.1020 17983310 10.1037/0278-7393.33.6.1020

[CR57] Unsworth, N. (2008). Exploring the retrieval dynamics of delayed and final free recall: Further evidence for temporal-contextual search. *Journal of Memory and Language,**59*(2), 223–236. 10.1016/j.jml.2008.04.002

[CR58] Unsworth, N. (2015). The influence of encoding manipulations on the dynamics of free recall. *Memory & Cognition,**43*(1), 60–69. 10.3758/s13421-014-0447-5 25030080 10.3758/s13421-014-0447-5

[CR59] Unsworth, N. (2019). Individual differences in long-term memory. *Psychological Bulletin,**145*(1), 79–139. 10.1037/bul0000176 30596433 10.1037/bul0000176

[CR60] Unsworth, N., & Brewer, G. A. (2010). Variation in working memory capacity and intrusions: Differences in generation or editing? *European Journal of Cognitive Psychology,**22*(6), 990–1000. 10.1080/09541440903175086

[CR61] Unsworth, N., Brewer, G. G. A., & Spillers, G. J. (2013). Working memory capacity and retrieval from long-term memory: the role of controlled search. *Memory & Cognition,**41*(2), 242–254. 10.3758/s13421-012-0261-x 23055120 10.3758/s13421-012-0261-x

[CR62] Unsworth, N., & Engle, R. W. (2007). The nature of individual differences in working memory capacity: Active maintenance in primary memory and controlled search from secondary memory. *Psychological Review,**114*(1), 104–132. 10.1037/0033-295X.114.1.104 17227183 10.1037/0033-295X.114.1.104

[CR63] Unsworth, N., Fukuda, K., Awh, E., & Vogel, E. K. (2014). Working memory and fluid intelligence: Capacity, attention control, and secondary memory retrieval. *Cognitive Psychology,**71*, 1–26. 10.1016/j.cogpsych.2014.01.003 24531497 10.1016/j.cogpsych.2014.01.003 PMC4484859

[CR64] Unsworth, N., Redick, T. S., Heitz, R. P., Broadway, J. M., & Engle, R. W. (2009). Complex working memory span tasks and higher-order cognition: a latent-variable analysis of the relationship between processing and storage. *Memory,**17*(6), 635–654. 10.1080/09658210902998047 19536691 10.1080/09658210902998047

[CR65] Unsworth, N., Spillers, G. J., & Brewer, G. A. (2012). Dynamics of context-dependent recall: An examination of internal and external context change. *Journal of Memory and Language,**66*(1), 1–16. 10.1016/j.jml.2011.05.001

[CR66] Unsworth, N., Spillers, G. J., Brewer, G., & a. (2010). The contributions of primary and secondary memory to working memory capacity: an individual differences analysis of immediate free recall. *Journal of Experimental Psychology: Learning, Memory, and Cognition,**36*(1), 240–247. 10.1037/a0017739 10.1037/a0017739 20053060

[CR67] Vergauwe, E., Camos, V., & Barrouillet, P. (2014). The impact of storage on processing: How is information maintained in working memory? *Journal of Experimental Psychology: Learning, Memory, and Cognition*, *40*(4). 10.1037/a0035779 10.1037/a0035779 24564542

[CR68] Ward, G., Tan, L., & Grenfell-Essam, R. (2010). Examining the relationship between free recall and immediate serial recall: The effects of list length and output order. *Journal of Experimental Psychology: Learning, Memory, and Cognition,**36*, 1207–1241. 20804293 10.1037/a0020122

[CR69] Wilhelm, O., Hildebrandt, A., & Oberauer, K. (2013). What is working memory capacity, and how can we measure it? *Frontiers in Psychology,**4*, 1–22. 10.3389/fpsyg.2013.00433 23898309 10.3389/fpsyg.2013.00433 PMC3721021

[CR70] Wixted, J. T., Ghadisha, H., & Vera, R. (1997). Recall Latency Following Pure-and Mixed-Strength Lists: A Direct Test of the Relative Strength Model of Free Recall. *Journal of Experimental Psychology: Learning, Memory, and Cognition,**23*(3), 523–538.

[CR71] Wixted, J. T., & Rohrer, D. (1993). Proactive interference and the dynamics of free recall. *Journal of Experimental Psychology: Learning, Memory, and Cognition,**19*(5), 1024–1039. 10.1037/0278-7393.19.5.1024

[CR72] Wixted, J. T., & Rohrer, D. (1994). Analyzing the dynamics of free recall: An integrative review of the empirical literature. *Psychonomic Bulletin & Review,**1*(1), 89–106. 10.3758/BF03200763 24203416 10.3758/BF03200763

[CR73] Zaromb, F. M., Howard, M. W., Dolan, E. D., Sirotin, Y. B., Tully, M., Wingfield, A., & Kahana, M. J. (2006). Temporal associations and prior-list intrusions in free recall. *Journal of Experimental Psychology: Learning Memory and Cognition,**32*(4), 792–804. 10.1037/0278-7393.32.4.792 16822147 10.1037/0278-7393.32.4.792

